# Blackbody-cavity ideal absorbers for solar energy harvesting

**DOI:** 10.1038/s41598-020-77372-9

**Published:** 2020-11-20

**Authors:** Yanpei Tian, Xiaojie Liu, Alok Ghanekar, Fangqi Chen, Andrew Caratenuto, Yi Zheng

**Affiliations:** 1 Department of Mechanical and Industrial Engineering, Northeastern University, Boston, MA USA; 2 Ming Hsieh Department of Electrical and Computer Engineering, University of Southern California, Los Angeles, CA 90089 USA; 3 Department of Electrical and Computer Engineering, Northeastern University, Boston, MA USA

**Keywords:** Mechanical engineering, Devices for energy harvesting

## Abstract

Spectrally selective solar absorbers (SSAs), which harvest heat from sunlight, are the key to concentrated solar thermal systems. An ideal SSA must have an absorptivity of unity in the solar irradiance wavelength region (0.3–2.5 $\upmu$m), and its infrared thermal emissivity must be zero to depress spontaneous blackbody irradiation (2.5–25 $\upmu$m). Current SSA designs which utilize photonic crystals, metamaterials, or cermets are either cost-inefficient due to the complexity of the required nanofabrication methods, or have limited applicability due to poor thermal stability at high temperatures. We conceptually present blackbody-cavity solar absorber designs with nearly ideal spectrally selective properties, capable of being manufactured at scale. The theoretical analyses show that the unity solar absorptivity of the blackbody cavity and nearly zero infrared emissivity of the SSA’s outer surface allow for a stagnation temperature of 880 $^\circ$C under 10 suns. The performance surpasses state-of-the-art SSAs manufactured using nanofabrication methods. This design relies only on traditional fabrication methods, such as machining, casting, and polishing. This makes it suitable for large-scale industrial applications, and the “blackbody cavity” feature enables easy integration with existing concentrated solar thermal systems using the parabolic reflector and Fresnel lens as optical concentrators.

## Introduction

**Figure 1:**
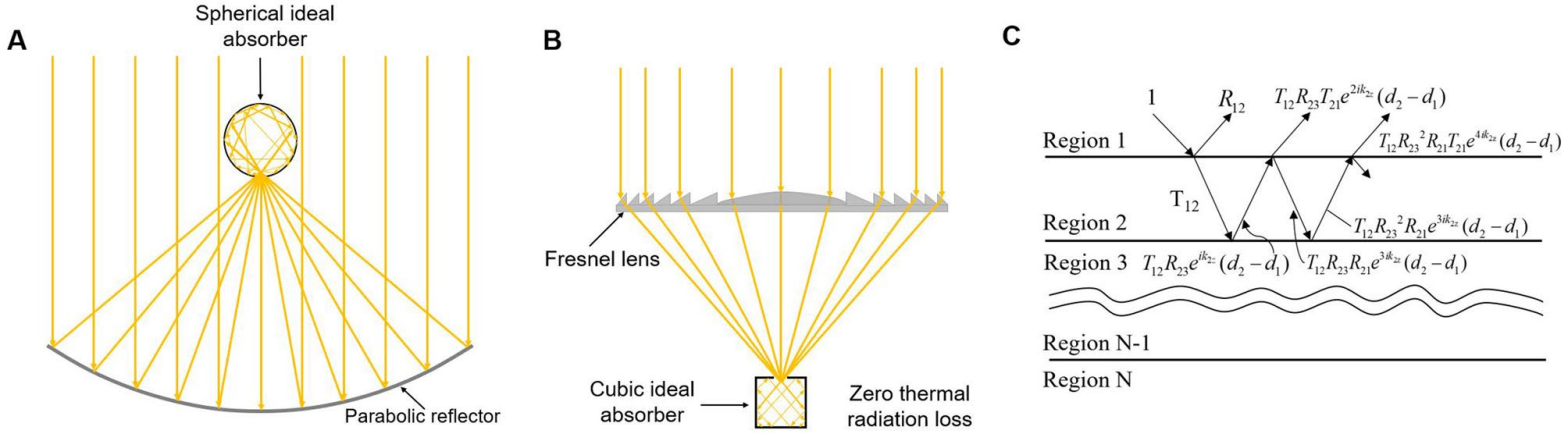
(**A**, **B**) Schematic of a blackbody cavity light absorbing system displaying the spherical (**A**) and cubic (**B**) BISAs integrated with the existing concentrated solar thermal systems using the parabolic reflector (**A**) and Fresnel lens (**B**) as optical concentrators, respectively. (**C**) Geometric series of multiple reflections in a N-Layer media.

Solar thermal technology is one of the most promising renewable energy options for replacing fossil fuels, as solar energy is the most abundant form of energy available on earth^1^. Recently, it has obtained increased attention in industrial engineering applications, such as heating, air conditioning, solar-driven desalination, and solar thermal electricity generation^2–6^. However, its large-scale industrial applications are impeded by the the relatively low solar-to-heat conversion efficiency, which arises from the thermal re-emission of blackbody radiation when the solar energy absorbing elements reach high temperatures. Solar absorbers, which convert solar radiation into heat, are a key component to the performance of various solar thermal systems, such as solar thermal power plants and solar thermoelectric generators, as well as solar thermophotovoltaics. Ideal SSAs possess a unity solar absorptivity to maximize solar heat gain, as well as a nearly zero infrared emissivity to minimize energy loss from spontaneous thermal radiation. SSAs composed of metamaterials^7–9^, cermets^10–12^, and photonic crystals^13–15^ have been extensively investigated in the past. However, these approaches often require stringent, complicated and time-consuming nanofabrication methods such as photolithography, chemical or physical vapor deposition, and etching. Furthermore, cost-effective scaling of such nanostructures is another hurdle in meeting the large scale requirements of potential industrial applications. Even if a simple and low-cost solution-based method is possible for large-scale fabrication of SSAs, the challenge of preparing a uniform and stable precursor containing as-designed metal ions still exists^16^. Therefore, simple, efficient, stable, and scalable approaches are desirable for commercial solar thermal systems. The feasible material candidates for SSAs are also confined to metals or ceramics^7,8,10,13,17^, such as Ni, W, Cr, Al$_2$O$_3$, SiO$_2$, and Cr$_2$O$_3$, which few fabrication technologies can handle. This affects the production as traditional large-scale fabrication techniques cannot easily be used. Additionally, although the aforementioned methods achieve good spectral selectivity, today’s state-of-the-art SSAs only have selective properties with 90% < $\alpha _{solar}$ < 95% and 3% < $\epsilon _{IR}$ < 10%^18^. Even an object with 3% $\epsilon _{IR}$ has a thermal radiation power density of $\sim$ 600 W/m$^2$ at 500 $^\circ$C, which causes high thermal losses and reduces system efficiency. Moreover, long-term thermal durability in a high-temperature operational environment is critical, since solar thermal engineering applications work under high concentrator factors in order to raise temperatures and increase efficiency. The mismatch in the thermal expansion coefficients of the metal and ceramic components, as well as the oxidation of the metal, will result in SSA fatigue and delamination after many high-temperature thermal cycles^19^. Single-element-based metal SSAs or widely-used metal alloy based SSAs could be a feasible candidate to address these challenges. Therefore, it is critical to fabricate SSAs with long-term durability through simple traditional fabrication procedures, such as machining, casting, soldering, and polishing. Here, we report several blackbody-cavity ideal solar absorbers (BISAs) based on the physical blackbody cavity model which have unity solar absorptivity and nearly zero infrared emissivity. Their manufacturing can be scaled to meet the requirements of large-scale industrial applications, such as those using the parabolic reflector and Fresnel lens based solar thermal systems (Fig. 1A,B). We simulate their thermal performances and achieve a stagnation temperature of 880 $^\circ$C under 10 suns, surpassing the state-of-the-art SSAs fabricated using nanofabrication methods. The potential materials for our designed SSAs can be single-element-based metals or metal alloys with a thermal coating that protects against oxidation. These materials can be easily manufactured by traditional approaches such as machining and polishing, which presents a cost-effective and time-saving alternative for a multitude of solar thermal engineering applications.

## Fundamental theory

### Theoretical fundamentals for the calculations of emissivity and reflectivity spectra

Now, let us consider a structure having *N*-layer media and having $(N-1)$ interfaces. The standard equation for the propagating transverse electric and magnetic waves can be used to describe electromagnetic fields in each medium (Fig. 1C). By solving for the boundary conditions at the interfaces, one can obtain the expression for the generalized reflection coefficient at the interface between region *i* and region $i+1$, which is given by^20^,

1 $$\begin{aligned} {\widetilde{R}}^{(\upmu )}_{i,i+1}=\frac{R^{(\upmu )}_{i,i+1}+{\widetilde{R}}^{(\upmu )}_{i+1,i+2}e^{2jk_{i+1,z}(d_{i+1}-d_{i})}}{1+R^{(\upmu )}_{i,i+1}{\widetilde{R}}^{(\upmu )}_{i+1,i+2}e^{2jk_{i+1,z}(d_{i+1}-d_{i})}} \end{aligned}$$

where $$R^{(\upmu )}_{i,i+r_{media1}}$$ is the Fresnel reflection coefficient at the interface between the layers *i* and $$i+1$$, $${\widetilde{R}}^{(\upmu )}_{i+1,i+2}$$ is the generalized reflection coefficient at the interface between the layers $$ i+1$$ and $$ i+2$$, $$\upmu =s$$ (or *p*) refers to transverse electric (or magnetic) polarization, and $$z=-d_{i}$$ is the location of the *i*th interface. $$k_{i,z}=\sqrt{\varepsilon _{i}(\omega )\omega ^{2}/c^{2}-k_{\rho }^{2}}$$ is the normal *z*-component of the wave vector in medium *i* wherein $$\varepsilon _{i}(\omega )$$ is the relative permittivity of the medium *i* as a function of angular frequency $$\omega $$, *c* is the speed of light in vacuum and $$k_{\rho } $$ is the magnitude of the in-plane wave vector. With $${\widetilde{R}}^{(\upmu )}_{N,N+1}=0$$, the above equation provides a recursive relation to calculate the reflection coefficients $${\widetilde{R}}^{(\upmu )}_{i,i+1}$$ in all regions. The generalized transmission coefficient for the layered slab is given by

2 $$\begin{aligned} {\widetilde{T}}^{(\upmu )}_{1,N}=\prod _{i=1}^{N-1}e^{jk_{iz}(d_{i}-d_{i-1})}S^{(\upmu )}_{i,i+1} \end{aligned}$$

where $S^{(\upmu )}_{i,i+1}\!=\!{T^{(\upmu )}_{i,i+1}}/{(1\!-\!R^{(\upmu )}_{i,i+1}{\widetilde{R}}^{(\upmu )}_{i+1,i+2}e^{2jk_{i+1,z}(d_{i+1}-d_{i})})}$. Alternatively, the generalized reflection and transmission coefficients can also be calculated using a transfer matrix approach^21^. The hemispherical emissivity is given by the expression

3 $$\begin{aligned} \epsilon (\omega )=\frac{c^{2}}{\omega ^{2}}\int _0^{\omega /c}dk_{\rho }k_{\rho }\sum _{\upmu =s,p}(1-|{\widetilde{R}}_{h1}^{(\upmu )}|^{2}-|{\widetilde{T}}_{h1}^{(\upmu )}|^{2}) \end{aligned}$$

where ${\widetilde{R}}_{h1}^{(\upmu )}$ and ${\widetilde{T}}_{h1}^{(\upmu )}$ are the polarized effective reflection and transmission coefficients.

### Energy efficiency, solar absorptivity, and thermal emissivity of SSAs

To quantitatively evaluate the performance of solar absorbers, the photon-to-heat conversion efficiency, $\eta _{abs}$, is given by:

4 $$\begin{aligned} \eta _{abs}=\alpha _{abs}-\epsilon _{abs} \frac{\sigma \left( T_{abs}^{4}-T_{amb}^{4}\right) }{C F \cdot Q_{abs}} \end{aligned}$$

where *CF* is the concentration factor, and $Q_{abs}$ is the solar radiative heat flux at AM 1.5 (global tilt)^22^. $\sigma$ is the Stefan-Boltzmann constant. $T_{abs}$ and $T_{amb}$ are the operational temperaturses of the solar absorber and the environment, respectively. The solar absorptance, $\alpha _{abs}$, is expressed as the following:

5 $$\begin{aligned} \alpha _{abs}=\frac{\int _{0.3 \upmu \mathrm{m}}^{4.0 \upmu \mathrm{m}} I_{sun}(\lambda ,\theta ,\phi ) \alpha (\lambda ,\theta , \phi ) d \lambda }{\int _{0.3 \upmu \mathrm{m}}^{4.0 \upmu \mathrm{m}} I_{sun}(\lambda ,\theta , \phi ) d \lambda }=\frac{\int _{0.3 \upmu \mathrm{m}}^{4.0 \upmu \mathrm{m}} I_{sun}(\lambda ,\theta , \phi )[1-R(\lambda ,\theta , \phi )] d \lambda }{\int _{0.3 \upmu \mathrm{m}}^{4.0 \upmu \mathrm{m}} I_{sun}(\lambda ,\theta , \phi ) d \lambda } \end{aligned}$$

where $\lambda$ is the wavelength of solar radiation, $\phi$ is the azimuthal angle, and $\theta$ is the polar angle. $\alpha (\lambda ,\theta , \phi )$ and $R(\lambda ,\theta , \phi )$ are the spectral directional absorptance and reflectance at a certain operational temperature. $I_{sun}$ is the incident solar intensity at AM 1.5 (global tilt)^22^. The numerator of this equation is the total absorbed solar energy, and the denominator is the incident solar heat flux, $Q_{abs}$. Since the available data of AM 1.5 is confined from 0.3 to 4.0 $\upmu$m^22^, which includes 99% of solar radiation, the integration interval is limited from 0.3 to 4.0 $\upmu$m. The total thermal emittance, $\epsilon _{abs}$, is given by:

6 $$\begin{aligned} \epsilon _{abs}=\frac{\int _{2.5 \upmu \mathrm{m}}^{20 \upmu \mathrm{m}} I_{bb}(\lambda ,\theta , \phi ) \epsilon (\lambda ,\theta , \phi ) d \lambda }{\int _{2.5 \upmu \mathrm{m}}^{20 \upmu \mathrm{m}} I_{bb}(\lambda ,\theta , \phi ) d \lambda }=\frac{\int _{2.5 \upmu \mathrm{m}}^{20 \upmu \mathrm{m}} I_{bb}(\lambda ,\theta , \phi )[1-R(\lambda ,\theta , \phi )] d \lambda }{\int _{2.5 \upmu \mathrm{m}}^{20 \upmu m} I_{bb}(\lambda ,\theta , \phi ) d \lambda } \end{aligned}$$

where $I_{bb}(\lambda ,\theta , \phi )$ is the blackbody radiation intensity given by Plank’s law. $\epsilon (\lambda ,\theta ,\phi )$ is the spectral directional absorptance at a certain operational temperature. Since our proposed structures are opaque within the wavelengths of interest (0.3–20 $\upmu$m), we take $\alpha (\lambda ,\theta , \phi )$ = $\epsilon (\lambda ,\theta , \phi )$ = 1 – $R(\lambda ,\theta , \phi )$ according to Kirchhoff’s law of thermal radiation.

### Investigations on the stagnation temperature under different concentration factors

The stagnation temperature of SSAs under different concentration factors, CF, can be solved by solving the thermal balance equation as expressed by:

7 $$\begin{aligned} \begin{aligned} Q_{total} =&Q_{sun} (T_{abs})+Q_{amb}(T_{amb}) -Q_{reemit} (T_{abs})-Q_{conv}(T_{abs}, T_{amb}) \end{aligned} \end{aligned}$$

The backsides of the SSAs are assumed to be thermally insulated. Here, *Q*$_{sun}$ is the heating power of the solar absorber from solar radiation. *Q*$_{amb}$ is incident thermal radiation from ambient, *Q*$_{reemit}$ is the heat flux through thermal re-emission from the solar absorber surface, *Q*$_{conv}$ is the convection energy transfer between solar absorbers and the ambient environment, and *Q*$_{total}$ is the net heating power of the solar absorber. *T*$_{abs}$ and *T*$_{amb}$ are the temperatures of solar absorbers and the ambient environment, respectively. *Q*$_{sun}$ can be determined as follows:

8 $$\begin{aligned} \begin{aligned} Q_{sun}\left( T_{abs}\right) =A \cdot CF \int _{0}^{\infty } d \lambda I_{AM 1.5}(\lambda ) \alpha _{abs}\left( \lambda , \theta _{sun}, T_{abs}\right) \end{aligned} \end{aligned}$$

where *A* is the total surface area of the solar absorber. $\lambda$ is the wavelength. $\alpha _{abs}(\lambda , \theta _{sun}, T_{abs})$ is the absorptivity of the solar absorber as a function of wavelength, the incident angle, and the temperature. The absorbed power from incident thermal radiation from the atmosphere $Q_{amb} (T_{amb})$ is given by:

9 $$\begin{aligned} \begin{aligned} Q_{amb}(T_{amb})&= A\cdot \int _0^\infty \mathrm{d}\lambda I_{BB} (T_{amb},\lambda )\epsilon (\lambda , \theta , \phi , T_{abs}) \epsilon (\lambda , \theta , \phi ) \end{aligned} \end{aligned}$$

where $I_{BB} (T_{abs}, \lambda )$ = $2hc^{2}{\lambda ^{-5}}$ $\exp ( hc/\lambda k_{B}T-1)^{-1}$ defines the spectral radiance of a blackbody at a certain temperature *T*, where *h* is Planck’s constant and $k_{B}$ is the Boltzmann constant. $\epsilon (\lambda , \theta , \phi , T_{abs})$ = $\frac{1}{\pi } \int _0^{2\pi }\mathrm{d}\phi \int _0^{\pi /2} \epsilon _{\lambda }\cos \theta \sin \theta \mathrm{d}\theta$ is the temperature-dependent emissivity of the solar absorber^23^. $\theta$ and $\phi$ are the azimuthal and latitudinal angles, respectively. The emissivity of the air, $\epsilon (\lambda , \theta , \phi )$, is given by $1-\tau (\lambda , \theta , \phi )$. Here, $\tau (\lambda , \theta , \phi )$ is the transmittance value of the atmosphere obtained from MODTRAN4^24^. $Q_{reemit}(T_{abs})$ is determined as follows:

10 $$\begin{aligned} Q_{reemit}(T_{abs}) = A\cdot \int _0^\infty \mathrm{d}\lambda I_{BB} (T_{abs}, \lambda ) \epsilon (\lambda , \theta , \phi , T_{abs}) \end{aligned}$$

Here, $I_{BB}(T_{BB}, \lambda )$ is the thermal radiation of a blackbody at a fixed temperature, $T_{abs}$. $\epsilon (\lambda , \theta , \phi , T_{abs})$ is the emissivity of the solar absorber according to Kirchhoff’s law of thermal radiation. $Q_conv(T_{abs}, T_{amb})$ is given by:

11 $$\begin{aligned} Q_{conv}(T_{amb}, T_{amb}) = A\cdot h_{a} \cdot (T_{abs}-T_{amb}) \end{aligned}$$

where $h_{a}$ is the convection coefficient of heat transfer between the solar absorber and the ambient environment. Here $h_{a}$ = 5 W m$^{-2}$ K$^{-1}$ is set as natural convection heat transfer to the solar absorber. The time-dependent temperature variations of the radiative cooler can be obtained by solving the following equation:

12 $$\begin{aligned} C_{abs} \frac{dT}{dt} = Q_{total}(T_{abs}, T_{amb}) \end{aligned}$$

For the solar absorber simulations, we assume the SSA to be a 5 mm $\times$ 5 mm $\times$ 5 mm tungsten cube with a thickness of 1 mm (the small hole area is negligible). The specific heat capacity of tungsten is 134 J/(kg $\cdot$ K), and its density is 19.25 g/cm$^3$ ($C_{abs} = \rho V C_{p}$). For more information on SSA simulations, see Ref. ^19^.

## Results

**Figure 2:**
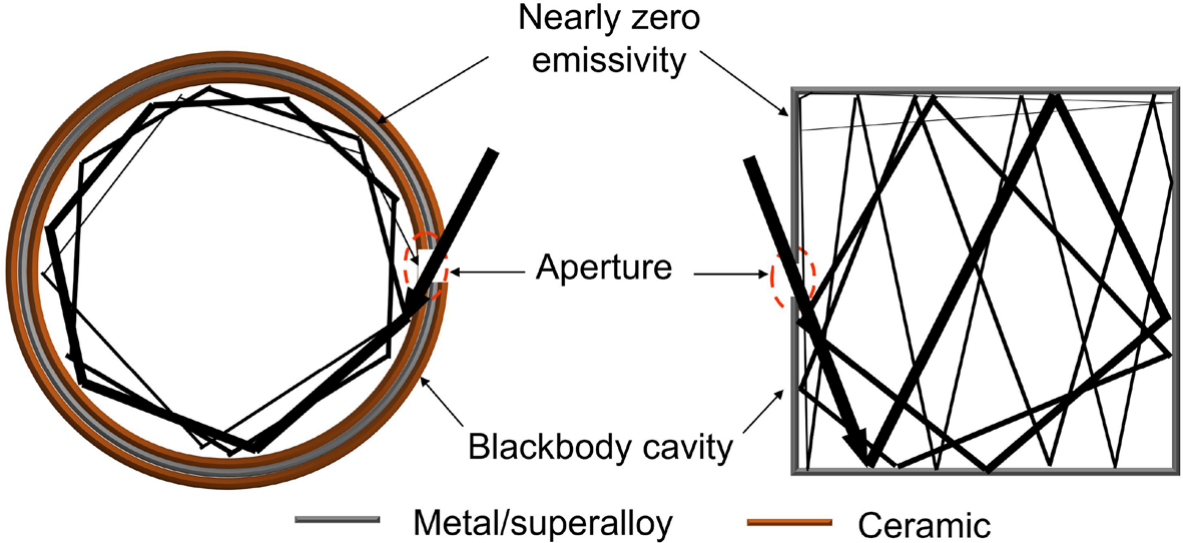
Left, ceramic sandwiched BISAs whose structures make them oxidation-resistant at high operational temperatures. Right, all-metal or superalloy-based BISAs.

**Figure 3:**
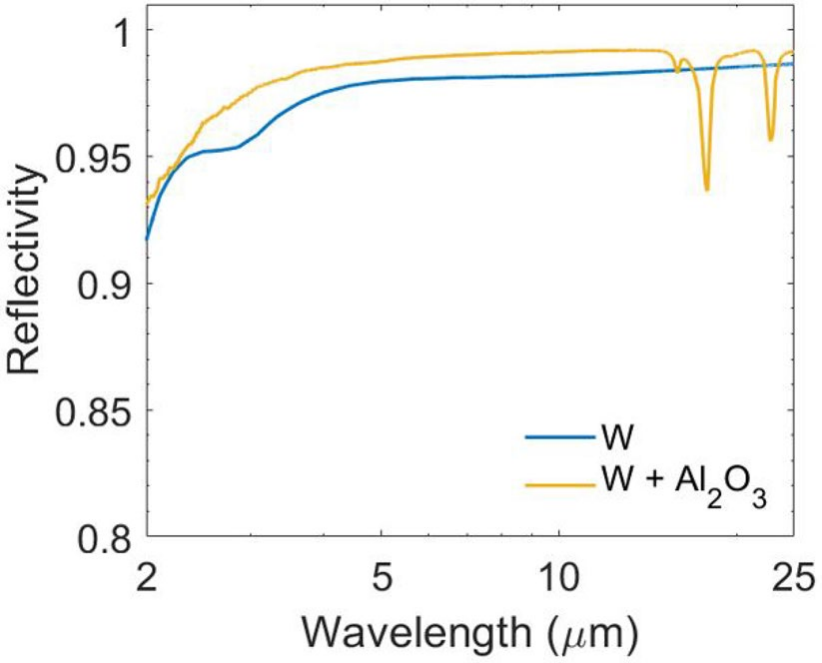
Spectral reflectivity of the W with and without a 100 nm thick ceramic (e.g. Al$_2$O$_3$) coating.

**Figure 4:**
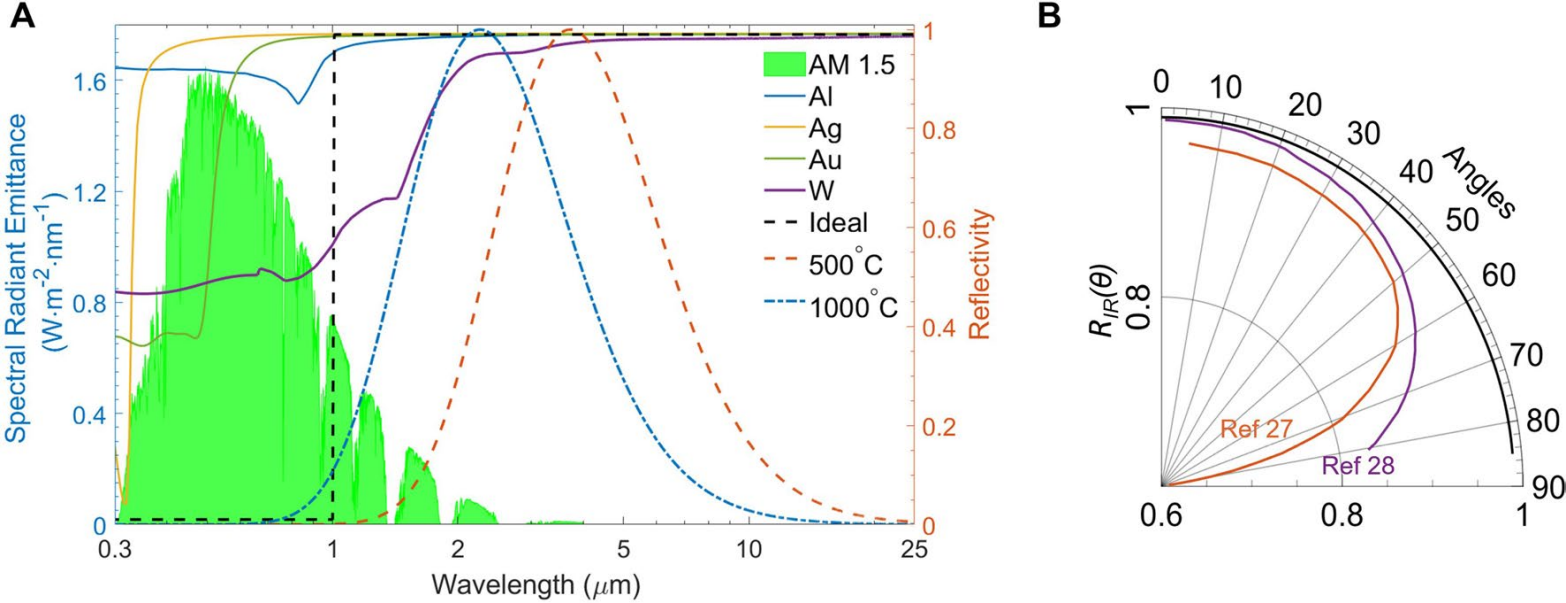
(**A**) Spectral reflectivity ($1-\epsilon$) of the Al, Ag, Au, W and ideal SSAs displayed with the ASTM G173 solar spectrum and the normalized blackbody irradiance at 500 $^\circ$C and 1000 $^\circ$C. (**B**) Near-unity reflectivity $R_{IR}$ across angles of BISAs’s external surface when fabricated by metal (here, W is used to calculate and average reflectivity from 1 to 25 $\upmu$m). The solar black curve represents the simulated angle-dependent thermal reflectivity of the 1 $\upmu$ mW, and the solid orange and purple curves depict the experimental results from Refs. ^27^ and ^28^, respectively.

**Figure 5:**
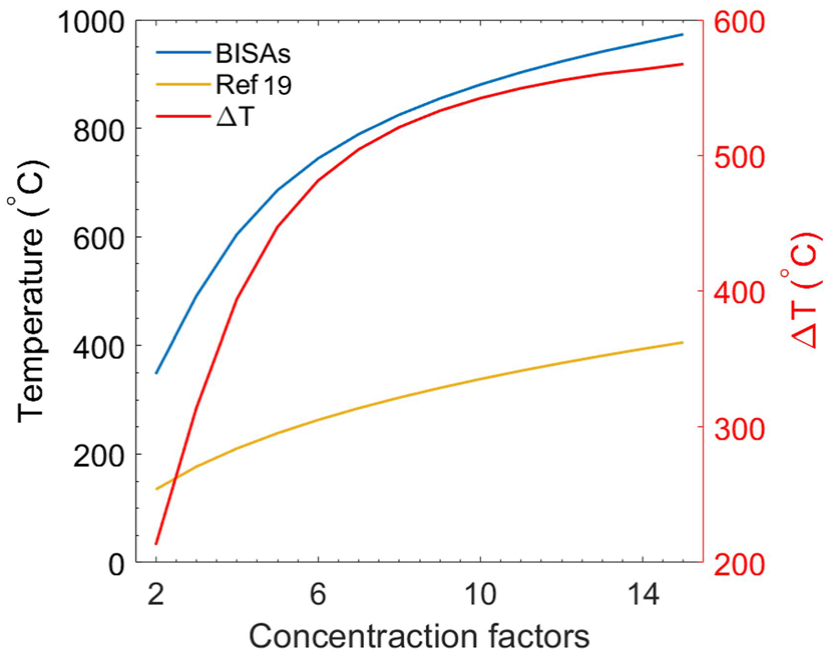
Left *y* axis: stagnation temperature as a function of concentration factors for BISAs and the cermet based SSAs fabricated by Cao et al.^17^ (the convection heat transfer coefficient, *h*, is 5 W/m$^2$ K). Right *y* axis: the difference of stagnation temperatures ($\Delta T$) between BISAs and the cermet based SSAs in Ref. ^14^.

When a spherical chamber with a small aperture is at thermal equilibrium, it can be regarded as an ideal blackbody^25^. The incident light undergoes reflection and absorption many times, and the light intensity decreases each time it contacts the internal surface and is partially absorbed. As such, very little light escapes from the spherical chamber. Therefore, the small cavity can be assumed to have the same properties as a blackbody. By increasing the absorptivity of the chamber material and fixing the area ratio ($\phi$) between the blackbody cavity area and the chamber surface area, the effective absorptivity of the blackbody becomes even larger. If $\phi$ < 0.6% and the internal surface absorptivity is 0.6, the overall absorptivity of the blackbody cavity is 0.996. In principle, the absorptivity of the chamber material does not affect the final absorptivity of the “blackbody cavity” (Fig. 2, left). That is, even if the material of the chamber is a highly reflective metal, such as Al, Ag, or W, the overall absorptivity of the blackbody cavity can still approach unity when an optimal area ratio is used. Generally, sunlight concentrates into beams with high energy density, which are absorbed by solar absorbers, but the incident angle of the concentrated beams is not always normal to the surface of solar absorbers. Therefore, the optical and thermal radiative properties of the solar absorber at oblique incident angles are vital for efficiently harvesting direct sunlight coming from various directions after reaching an optical concentrator^7^. The absorptivity of the blackbody cavity is also independent of the incident angle since the incident light from any angle will be absorbed by the internal surface of the chamber. The shape of the blackbody chamber can also be cubic (Fig. 2, right), cylindrical, or conical, as well as any other shape according to the demand for a practical application, since the only parameter that affects the solar absorptivity is the area ratio. The BISAs can be fabricated directly using metal since the metals have nearl-unity reflectivity in the infrared range, enabling the outer surface of the blackbody chamber to have barely any thermal losses due to spontaneous thermal radiation. The metals can also be sandwich-coated with ceramic materials (e.g. SiO$_2$, Al$_2$O$_3$, etc.) or replaced with superalloys (e.g. Inconel, Waspaloy, etc.) to become oxidation-resistant for their use in a high-temperature environment (high concentration factors). For example, 100 nm thick Al$_2$O$_3$ coated with W shows little change and even an increased reflectivity from 2 to 25 $\upmu$m (Fig. 3A) (calculation approaches of reflectivity spectra are provided in the fundamental theory section^20,21,26^). Additionally, the concentrating optics used in concentrated solar thermal systems converge the incident sunlight to a small point, which it makes the blackbody cavity a perfect candidate for integration into to existing solar thermal systems using the Fresnel lens or the parabolic reflector as concentrating optics without complex modifications (Fig. 1A,B). Figure 4A shows that some common metals, like Al, Ag, Au, Ni, and W, have near-unity reflectivity in the thermal infrared region (2–25 $\upmu$m). Consequently, these metals or related alloys can be selected as alternative materials for different temperature applications according to their melting and oxidation temperatures. Furthermore, metals such as W show an excellent reflectivity in the infrared region across the incident angle (0°–85°) that is higher than previously reported values (Fig. 4B)^27,28^. The novel design of BISAs results in spectral selectivity that approaches the ideal case, exemplified by a stagnation temperature of 880 $^\circ$C under a concentration factor of 10 (10 $\times$ AM 1.5 solar intensity). It is 542 $^\circ$C higher than the stagnation temperature of cermet based SSAs (the reflectivity spectra used in the calculation of stagnation temperature are taken from Ref. ^19^. Details about the stagnation temperature calculation relevant to Fig. 5 are provided in the fundamental theory section).

**Table 1:** Comparison of the SSAs (photonic crystals, metamaterials, and cermet).

	Materials^a^	Substrate^a^	Fabrication methods^a^	Thermal stability	Solar absorptivity	Thermal emissivity
Meta-materials^b^	SiO$_2$ filled 2D W nanohole + SiO$_2$ spacer^29^	W	PECVD+RIE in air	3 h 1000 $^\circ$C	0.9	0.23 (800 $^\circ$C)
	2D Ti gratings + MgF$_2$ spacer^7^	W	e-beam evaporation+e-beam lithography	350 $^\circ$C in air	0.9	0.2(25 $^\circ$C)
	Ti triangular nanodisks +Al$_2$O$_3$ spacer^9^	Ta	e-beam lithography	5 h 727 $^\circ$C in air	0.91	0.24 (1000 $^\circ$C)
Cermet-based^b^	Cr (inclusions) + Cr$_2$O$_3$ (matrix)^30^	SS	Sputtering	300 $^\circ$C	0.92	0.08 (121 $^\circ$C)
	Ni (inclusions) + Cr$_2$O$_3$ (matrix)^31^	Fused quartz	Evaporation	500 $^\circ$C in air	0.94	0.1
	W (inclusions) + AlN (matrix)^32^	Glass	Sputtering	500 $^\circ$C in vacuum	0.939	0.039 (27 $^\circ$C)
	Ni (inclusions) + SiO$_2$ (matrix)^33^	Quartz	Evaporation	500 $^\circ$C in vacuum	0.9	0.07 (100 $^\circ$C)
BISAs^c^	Metal/superalloy	−	Machining/casting + polishing	–	$\sim$ 1	$\sim$ 1

^a^SiO$_2$: Silicon dioxide; W: Tungsten; Ti: Titanium; MgF$_2$: Magnesium fluoride; Al$_2$O$_3$: Aluminium oxide; Cr: Chromium; Cr$_2$O$_3$: Chromium(III) oxide; Ni: Nickel; AlN: Aluminium nitride; Ta: Tantalum; SS: Stainless steel; PECVD: Plasma-enhanced chemical vapor deposition; RIE:Reactive-ion etching.

^b^Experimental results.

^c^Simulated results.

## Discussion

To summarize, we demonstrate nearly perfect SSAs with unity solar absorptivity and nearly zero infrared thermal emissivity by employing a novel blackbody cavity thermal radiation model. The common metamaterial- and cermet-based SSAs both use a metal-dielectric structure on top of the metal or dielectric substrate, which undergoes thermal fatigue and delamination as the number of high-temperature working cycles increases. The nanometer features created via complicated and cost-ineffective nanofabrication methods cause thermal fatigue to become even more severe. As listed in Table 1, the best reported thermal stability performance of a metamaterial- or cermet-based SSA is 3 h at 1000 $^\circ$C, which still cannot meet the requirements of concentrated solar thermal engineering. However, our proposed BISAs offer a great alternative to these designs, since the superalloy has been validated to work under even higher temperatures and for longer times^34^. The proposed BISAs can also absorb the light beams from different angles behind the optical concentrators and thus increase the solar energy conversion efficiency, since its solar absorptivity of the BISAs is angle-independent. Additionally, we demonstrate a stagnation temperature of 880 $^\circ$C under 10 suns. The BISAs can be fabricated by machining, casting, or soldering chosen metals or superalloys into different shapes, followed by the polishing of their external surfaces into a highly reflective “mirror” to suppress the energy loss from spontaneous thermal radiation. The polished tungsten is not wear-resistant. If the polished outer surface is worn, its thermal reflectivity will decrease, and the stagnation temperature drops simultaneously. Whether the stagnation temperature drops rapidly or smoothly depends on the degree of wear. Existing concentrated solar thermal systems are mostly based on the parabolic reflector, Fresnel lens, or tracking flat mirror arrays, which concentrate the sunlight into a light beam with high energy density. Therefore, the blackbody cavity can easily be used as an entrance port for the sunlight beams, and the inner surface of the chamber will absorb almost all the solar energy and transfer it to the working fluid. This novel design of an absorbing blackbody cavity, able to be manufactured at scale, is highly suited to be integrated into existing concentrated solar thermal systems.
